# *t*-3-Benzyl-*r*-2,*c*-6-bis­(4-methoxy­phen­yl)­piperidin-4-one

**DOI:** 10.1107/S1600536808015717

**Published:** 2008-06-07

**Authors:** J. Jayabharathi, A. Thangamani, S. Balamurugan, A. Thiruvalluvar, A. Linden

**Affiliations:** a Department of Chemistry, Annamalai University, Annamalai Nagar 608 002, Tamil Nadu, India; b PG Research Department of Physics, Rajah Serfoji Government College (Autonomous), Thanjavur 613 005, Tamil Nadu, India; c Institute of Organic Chemistry, University of Zürich, Winterthurerstrasse 190, CH-8057 Zürich, Switzerland

## Abstract

In the title compound, C_26_H_27_NO_3_, the piperidine ring adopts a chair conformation. The two methoxy­phenyl groups attached to the piperidine ring at positions 2 and 6 have equatorial orientations and make a dihedral angle of 87.33 (8)°. The benzyl group at position 3 has an equatorial orientation. The phenyl ring of the benzyl group makes dihedral angles of 75.60 (9) and 73.69 (9)° with the two benzene rings. Mol­ecules are linked by inter­molecular N—H⋯O and C—H⋯O hydrogen bonds and by C—H⋯π inter­actions.

## Related literature

For related literature, see: Jayabharathi *et al.* (2007 ▶); Thiruvalluvar *et al.* (2007 ▶); Noller & Baliah (1948 ▶).
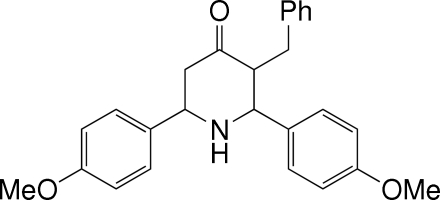


## Experimental

### 

#### Crystal data

C_26_H_27_NO_3_
*M* *_r_* = 401.49
Triclinic, 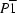

*a* = 5.5612 (2) Å
*b* = 13.8097 (5) Å
*c* = 14.5119 (6) Å
α = 71.629 (2)°
β = 79.928 (2)°
γ = 82.526 (2)°
*V* = 1038.04 (7) Å^3^
*Z* = 2
Mo *K*α radiation
μ = 0.08 mm^−1^
*T* = 160 (1) K
0.30 × 0.18 × 0.13 mm

#### Data collection

Nonius KappaCCD area-detector diffractometer
Absorption correction: none
25390 measured reflections
4740 independent reflections
3325 reflections with *I* > 2σ(*I*)
*R*_int_ = 0.052

#### Refinement

*R*[*F* ^2^ > 2σ(*F* ^2^)] = 0.053
*wR*(*F* ^2^) = 0.177
*S* = 1.09
4740 reflections
275 parameters
H atoms treated by a mixture of independent and constrained refinement
Δρ_max_ = 0.28 e Å^−3^
Δρ_min_ = −0.32 e Å^−3^

### 

Data collection: *COLLECT* (Nonius, 2000 ▶); cell refinement: *DENZO-SMN* (Otwinowski & Minor, 1997 ▶); data reduction: *DENZO-SMN* and *SCALEPACK* (Otwinowski & Minor, 1997 ▶); program(s) used to solve structure: *SHELXS97* (Sheldrick, 2008 ▶); program(s) used to refine structure: *SHELXL97* (Sheldrick, 2008 ▶); molecular graphics: *ORTEP-3* (Farrugia, 1997 ▶); software used to prepare material for publication: *PLATON* (Spek, 2003 ▶).

## Supplementary Material

Crystal structure: contains datablocks global, I. DOI: 10.1107/S1600536808015717/wn2265sup1.cif

Structure factors: contains datablocks I. DOI: 10.1107/S1600536808015717/wn2265Isup2.hkl

Additional supplementary materials:  crystallographic information; 3D view; checkCIF report

## Figures and Tables

**Table 1:** Hydrogen-bond geometry (Å, °)

*D*—H⋯*A*	*D*—H	H⋯*A*	*D*⋯*A*	*D*—H⋯*A*
N1—H1⋯O2^i^	0.92 (2)	2.56 (2)	3.420 (2)	154.9 (14)
C5—H5*B*⋯O4^ii^	0.99	2.51	3.490 (2)	173
C16—H16*B*⋯*Cg*1^iii^	0.98	2.85	3.659 (2)	140
C16—H16*C*⋯*Cg*2^iv^	0.98	2.98	3.587 (2)	121
C34—H34⋯*Cg*3^v^	0.95	2.91	3.719 (2)	144

Symmetry codes: (i) 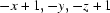; (ii) 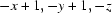; (iii) 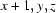; (iv) 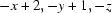; (v) 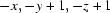. *Cg*1, *Cg*2 and *Cg*3 are the centroids of the C61–C66, C31–C36 and C21–26 rings, respectively.

**Fig. 1.**
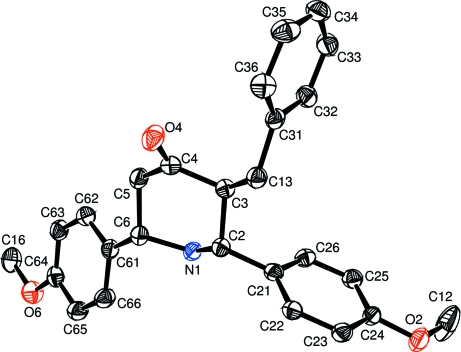
The molecular structure of the title compound, showing the atom-numbering scheme and displacement ellipsoids drawn at the 50% probability level. H atoms have been omitted for clarity.

**Fig. 2.**
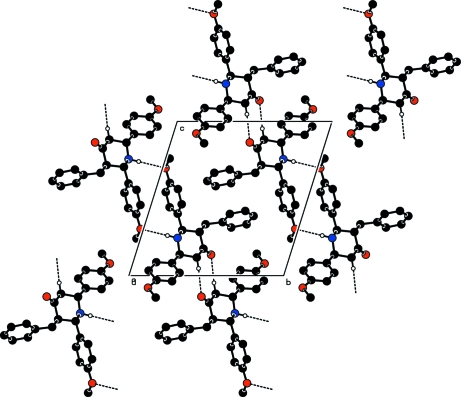
The molecular packing of the title compound, viewed down the a axis. Dashed lines indicate hydrogen bonds. H atoms not involved in hydrogen bonding have been omitted.

## supplementary crystallographic information

### Comment

Jayabharathi *et al.* (2007) have reported the synthesis, stereochemistry and antimicrobial evaluation of t3-benzyl-r2,c6-diarylpiperidin- 4-one and its derivatives. Thiruvalluvar *et al.* (2007) have reported the crystal structure of t3-benzyl-1-formyl-r2,c-6-diphenylpiperidin-4-one, in which the piperidine ring is in a distorted boat form.

In the title compound, (Fig. 1), the piperidine ring adopts a chair conformation. The two methoxyphenyl groups attached to the piperidine ring at positions 2 and 6 have equatorial orientations, and make a dihedral angle of 87.33 (8)°. The benzyl group at position 3 has an equatorial orientation. The phenyl ring of the benzyl group makes a dihedral angle of 75.60 (9)° with the benzene ring at C2, and 73.69 (9)° with the benzene ring at C6. Molecules are linked by intermolecular N1—H1···O2 and C5—H5B···O4 hydrogen bonds (Fig. 2). There are C16—H16B···π (1 + *x*, *y*, *z*) interactions involving the benzene ring at C6, C16—H16C···π (2 - *x*, 1 - *y*, -*z*) interactions involving the phenyl ring at C13 and C34—H34···π (-*x*, 1 - *y*, 1 - *z*) interactions involving the benzene ring at C2.

### Experimental

The title compound was prepared according to the literature procedure (Noller & Baliah, 1948). A mixture of ammonium acetate (0.1 mol, 7.7 g), redistilled 4-methoxybenzaldehyde (0.2 mol, 24.3 ml) and benzylacetone (0.1 mol, 15 ml) in distilled ethanol was heated to boiling. The mixture was cooled and 10 ml hydrochloric acid was added. The precipitated 3-benzyl-r2,c6-bis(*p*-methoxyphenyl)piperidin-4-one hydrochlorides were treated with aqueous ammonia and diluted with water. The separated solid was filtered off and recrystallized from ethanol. The yield of the isolated pure product was 10.5 g (70%).

### Refinement

The H atom bonded to N1 was located in a difference Fourier map and refined isotropically to an N—H bond length of 0.92 (2) Å. The remaining H atoms were positioned geometrically and allowed to ride on their parent atoms, with C—H = 0.95–1.00 Å and *U*_iso_(H) = *xU*_eq_(carrier atom), where *x* = 1.5 for methyl and 1.2 for all other C atoms.

### Crystal data

**Table:**

C_26_H_27_NO_3_	*Z* = 2
*M**_r_* = 401.49	*F*_000_ = 428
Triclinic, *P*1	*D*_x_ = 1.285 Mg m^−^^3^
Hall symbol: -P 1	Melting point: 471 K
*a* = 5.5612 (2) Å	Mo *K*α radiation λ = 0.71073 Å
*b* = 13.8097 (5) Å	Cell parameters from 4566 reflections
*c* = 14.5119 (6) Å	θ = 2.0–27.5º
α = 71.629 (2)º	µ = 0.08 mm^−^^1^
β = 79.928 (2)º	*T* = 160 (1) K
γ = 82.526 (2)º	Prism, colourless
*V* = 1038.04 (7) Å^3^	0.30 × 0.18 × 0.13 mm

### Data collection

**Table:**

Nonius KappaCCD area-detector diffractometer	4740 independent reflections
Radiation source: Nonius FR590 sealed tube generator	3325 reflections with *I* > 2σ(*I*)
Monochromator: horizontally mounted graphite crystal	*R*_int_ = 0.052
Detector resolution: 9 pixels mm^-1^	θ_max_ = 27.5º
*T* = 160(1) K	θ_min_ = 3.0º
φ and ω scans with κ offsets	*h* = −7→7
Absorption correction: none	*k* = −17→17
25390 measured reflections	*l* = −17→18

### Refinement

**Table:**

Refinement on *F*^2^	Secondary atom site location: difference Fourier map
Least-squares matrix: full	Hydrogen site location: inferred from neighbouring sites
*R*[*F*^2^ > 2σ(*F*^2^)] = 0.053	H atoms treated by a mixture of independent and constrained refinement
*wR*(*F*^2^) = 0.177	*w* = 1/[σ^2^(*F*_o_^2^) + (0.1039*P*)^2^ + 0.0262*P*] where *P* = (*F*_o_^2^ + 2*F*_c_^2^)/3
*S* = 1.09	(Δ/σ)_max_ < 0.001
4740 reflections	Δρ_max_ = 0.28 e Å^−^^3^
275 parameters	Δρ_min_ = −0.32 e Å^−^^3^
Primary atom site location: structure-invariant direct methods	Extinction correction: none

### Special details

**Table:**

Experimental. Solvent used:Ethanol Cooling Device: Oxford Cryosystems Cryostream 700 Crystal mount: glued on a glass fibre Mosaicity (°.): 0.788 (2) Frames collected: 372 Seconds exposure per frame: 40 Degrees rotation per frame: 2.0 Crystal-Detector distance (mm): 32.0
Geometry. Bond distances, angles *etc*. have been calculated using the rounded fractional coordinates. All su's are estimated from the variances of the (full) variance-covariance matrix. The cell e.s.d.'s are taken into account in the estimation of distances, angles and torsion angles
Refinement. Refinement of *F*^2^ against ALL reflections. The weighted *R*-factor *wR* and goodness of fit *S* are based on *F*^2^, conventional *R*-factors *R* are based on *F*, with *F* set to zero for negative *F*^2^. The threshold expression of *F*^2^ > σ(*F*^2^) is used only for calculating *R*-factors(gt) *etc*. and is not relevant to the choice of reflections for refinement. *R*-factors based on *F*^2^ are statistically about twice as large as those based on *F*, and *R*- factors based on ALL data will be even larger.

### Fractional atomic coordinates and isotropic or equivalent isotropic displacement parameters (Å^2^)

**Table:**

	*x*	*y*	*z*	*U*_iso_*/*U*_eq_	
O2	0.3480 (3)	0.01839 (10)	0.69755 (9)	0.0380 (4)	
O4	0.2802 (3)	0.48862 (10)	0.13605 (9)	0.0404 (4)	
O6	1.5838 (2)	0.14802 (10)	−0.07836 (10)	0.0409 (5)	
N1	0.7126 (3)	0.23805 (11)	0.24454 (10)	0.0265 (5)	
C2	0.4629 (3)	0.25237 (12)	0.29523 (12)	0.0245 (5)	
C3	0.4009 (3)	0.36832 (12)	0.28311 (12)	0.0250 (5)	
C4	0.4384 (3)	0.42694 (12)	0.17505 (12)	0.0279 (5)	
C5	0.6790 (3)	0.40193 (13)	0.11763 (12)	0.0301 (5)	
C6	0.7249 (3)	0.28549 (13)	0.13751 (12)	0.0273 (5)	
C12	0.5115 (5)	0.0257 (2)	0.75990 (16)	0.0691 (10)	
C13	0.1459 (3)	0.39167 (13)	0.33331 (13)	0.0279 (5)	
C16	1.7542 (4)	0.21784 (17)	−0.14188 (14)	0.0428 (7)	
C21	0.4411 (3)	0.18956 (12)	0.40212 (12)	0.0249 (5)	
C22	0.2541 (3)	0.12454 (12)	0.44361 (12)	0.0262 (5)	
C23	0.2270 (3)	0.06881 (12)	0.54173 (12)	0.0278 (5)	
C24	0.3891 (3)	0.07709 (12)	0.60121 (12)	0.0267 (5)	
C25	0.5762 (3)	0.14155 (13)	0.56151 (13)	0.0287 (5)	
C26	0.6007 (3)	0.19672 (12)	0.46278 (12)	0.0271 (5)	
C31	0.0983 (3)	0.49710 (12)	0.34677 (12)	0.0258 (5)	
C32	0.2424 (3)	0.52745 (13)	0.40038 (13)	0.0300 (6)	
C33	0.1909 (4)	0.62079 (14)	0.41958 (13)	0.0346 (6)	
C34	−0.0083 (4)	0.68498 (14)	0.38666 (14)	0.0366 (6)	
C35	−0.1530 (3)	0.65645 (14)	0.33296 (15)	0.0380 (6)	
C36	−0.0982 (3)	0.56370 (14)	0.31217 (14)	0.0333 (6)	
C61	0.9614 (3)	0.25342 (13)	0.08147 (12)	0.0274 (5)	
C62	1.1216 (3)	0.32162 (14)	0.01856 (13)	0.0317 (6)	
C63	1.3309 (3)	0.29035 (14)	−0.03643 (13)	0.0326 (6)	
C64	1.3824 (3)	0.18795 (14)	−0.02915 (13)	0.0303 (6)	
C65	1.2233 (4)	0.11740 (14)	0.03294 (14)	0.0356 (6)	
C66	1.0172 (3)	0.14985 (14)	0.08710 (13)	0.0332 (6)	
H1	0.750 (3)	0.1682 (16)	0.2598 (14)	0.033 (5)*	
H2	0.34531	0.22921	0.26323	0.0294*	
H3	0.52128	0.39034	0.31473	0.0300*	
H5A	0.81371	0.42810	0.13704	0.0361*	
H5B	0.67513	0.43557	0.04667	0.0361*	
H6	0.58725	0.26183	0.11619	0.0328*	
H12A	0.46528	−0.01939	0.82636	0.1039*	
H12B	0.50264	0.09654	0.76144	0.1039*	
H12C	0.67912	0.00504	0.73486	0.1039*	
H13A	0.02410	0.38391	0.29437	0.0335*	
H13B	0.11882	0.34008	0.39858	0.0335*	
H16A	1.88915	0.17995	−0.17237	0.0641*	
H16B	1.81921	0.25311	−0.10372	0.0641*	
H16C	1.67091	0.26824	−0.19301	0.0641*	
H22	0.14246	0.11832	0.40355	0.0314*	
H23	0.09773	0.02490	0.56854	0.0333*	
H25	0.68732	0.14796	0.60169	0.0344*	
H26	0.73009	0.24055	0.43600	0.0325*	
H32	0.37858	0.48344	0.42428	0.0360*	
H33	0.29303	0.64058	0.45548	0.0415*	
H34	−0.04559	0.74834	0.40085	0.0439*	
H35	−0.29042	0.70032	0.31013	0.0455*	
H36	−0.19660	0.54560	0.27376	0.0400*	
H62	1.08791	0.39239	0.01259	0.0380*	
H63	1.43764	0.33931	−0.07879	0.0391*	
H65	1.25646	0.04680	0.03805	0.0426*	
H66	0.91040	0.10079	0.12928	0.0398*	

### Atomic displacement parameters (Å^2^)

**Table:**

	*U*^11^	*U*^22^	*U*^33^	*U*^12^	*U*^13^	*U*^23^
O2	0.0459 (8)	0.0394 (8)	0.0250 (7)	−0.0122 (6)	−0.0048 (6)	−0.0012 (6)
O4	0.0440 (8)	0.0385 (8)	0.0300 (7)	0.0116 (6)	−0.0047 (6)	−0.0042 (6)
O6	0.0350 (8)	0.0445 (8)	0.0406 (8)	−0.0017 (6)	0.0080 (6)	−0.0169 (7)
N1	0.0293 (8)	0.0239 (8)	0.0234 (8)	−0.0005 (6)	−0.0018 (6)	−0.0045 (6)
C2	0.0257 (9)	0.0248 (9)	0.0228 (8)	−0.0035 (7)	−0.0020 (7)	−0.0069 (7)
C3	0.0257 (9)	0.0245 (9)	0.0248 (9)	−0.0025 (7)	−0.0035 (7)	−0.0071 (7)
C4	0.0333 (10)	0.0226 (9)	0.0275 (9)	−0.0031 (7)	−0.0034 (8)	−0.0073 (7)
C5	0.0342 (10)	0.0293 (9)	0.0229 (9)	−0.0009 (8)	−0.0003 (7)	−0.0051 (7)
C6	0.0304 (10)	0.0280 (9)	0.0227 (9)	−0.0022 (7)	−0.0026 (7)	−0.0070 (7)
C12	0.0737 (18)	0.098 (2)	0.0292 (11)	−0.0421 (15)	−0.0179 (12)	0.0097 (12)
C13	0.0279 (10)	0.0281 (9)	0.0265 (9)	−0.0027 (7)	−0.0018 (7)	−0.0075 (7)
C16	0.0341 (11)	0.0567 (13)	0.0335 (11)	−0.0044 (9)	0.0053 (9)	−0.0130 (10)
C21	0.0264 (9)	0.0209 (8)	0.0269 (9)	0.0004 (7)	−0.0019 (7)	−0.0084 (7)
C22	0.0282 (9)	0.0240 (9)	0.0277 (9)	−0.0032 (7)	−0.0034 (7)	−0.0093 (7)
C23	0.0277 (10)	0.0257 (9)	0.0297 (9)	−0.0066 (7)	0.0004 (7)	−0.0085 (7)
C24	0.0314 (10)	0.0227 (9)	0.0228 (8)	−0.0007 (7)	−0.0006 (7)	−0.0046 (7)
C25	0.0302 (10)	0.0264 (9)	0.0298 (9)	−0.0019 (7)	−0.0065 (7)	−0.0078 (7)
C26	0.0251 (9)	0.0241 (9)	0.0303 (9)	−0.0040 (7)	−0.0021 (7)	−0.0060 (7)
C31	0.0242 (9)	0.0259 (9)	0.0233 (8)	−0.0027 (7)	0.0031 (7)	−0.0051 (7)
C32	0.0284 (10)	0.0315 (10)	0.0288 (9)	0.0007 (7)	−0.0006 (7)	−0.0101 (8)
C33	0.0383 (11)	0.0351 (10)	0.0317 (10)	−0.0065 (8)	0.0011 (8)	−0.0135 (8)
C34	0.0406 (11)	0.0273 (9)	0.0400 (11)	−0.0034 (8)	0.0073 (9)	−0.0142 (8)
C35	0.0299 (10)	0.0301 (10)	0.0483 (12)	0.0038 (8)	−0.0016 (9)	−0.0085 (9)
C36	0.0276 (10)	0.0346 (10)	0.0376 (10)	−0.0019 (8)	−0.0040 (8)	−0.0111 (8)
C61	0.0298 (10)	0.0292 (9)	0.0225 (8)	−0.0010 (7)	−0.0046 (7)	−0.0067 (7)
C62	0.0357 (11)	0.0293 (9)	0.0289 (10)	−0.0019 (8)	−0.0018 (8)	−0.0090 (8)
C63	0.0337 (10)	0.0360 (10)	0.0260 (9)	−0.0078 (8)	0.0017 (8)	−0.0075 (8)
C64	0.0290 (10)	0.0374 (10)	0.0252 (9)	0.0012 (8)	−0.0025 (7)	−0.0125 (8)
C65	0.0412 (11)	0.0281 (9)	0.0349 (10)	−0.0006 (8)	−0.0006 (8)	−0.0093 (8)
C66	0.0342 (10)	0.0292 (9)	0.0317 (10)	−0.0036 (8)	0.0040 (8)	−0.0071 (8)

### Geometric parameters (Å, °)

**Table:**

O2—C12	1.423 (3)	C62—C63	1.393 (3)
O2—C24	1.373 (2)	C63—C64	1.380 (3)
O4—C4	1.223 (2)	C64—C65	1.391 (3)
O6—C16	1.432 (3)	C65—C66	1.380 (3)
O6—C64	1.370 (2)	C2—H2	1.0000
N1—C2	1.474 (2)	C3—H3	1.0000
N1—C6	1.476 (2)	C5—H5A	0.9900
N1—H1	0.92 (2)	C5—H5B	0.9900
C2—C3	1.554 (2)	C6—H6	1.0000
C2—C21	1.512 (2)	C12—H12A	0.9800
C3—C4	1.512 (2)	C12—H12B	0.9800
C3—C13	1.521 (2)	C12—H12C	0.9800
C4—C5	1.508 (2)	C13—H13A	0.9900
C5—C6	1.536 (3)	C13—H13B	0.9900
C6—C61	1.513 (2)	C16—H16A	0.9800
C13—C31	1.513 (3)	C16—H16B	0.9800
C21—C26	1.387 (2)	C16—H16C	0.9800
C21—C22	1.391 (2)	C22—H22	0.9500
C22—C23	1.381 (2)	C23—H23	0.9500
C23—C24	1.390 (2)	C25—H25	0.9500
C24—C25	1.384 (2)	C26—H26	0.9500
C25—C26	1.387 (2)	C32—H32	0.9500
C31—C32	1.391 (2)	C33—H33	0.9500
C31—C36	1.390 (3)	C34—H34	0.9500
C32—C33	1.387 (3)	C35—H35	0.9500
C33—C34	1.380 (3)	C36—H36	0.9500
C34—C35	1.380 (3)	C62—H62	0.9500
C35—C36	1.391 (3)	C63—H63	0.9500
C61—C66	1.403 (3)	C65—H65	0.9500
C61—C62	1.381 (2)	C66—H66	0.9500
			
O4···C36	3.355 (2)	H3···C26	2.8900
O4···C31	3.082 (2)	H3···C32	2.7500
O6···C12^i^	3.396 (3)	H3···H26	2.5500
O2···H1^ii^	2.56 (2)	H3···H32	2.3200
O2···H66^ii^	2.8000	H5A···O4^xii^	2.8200
O4···H5A^iii^	2.8200	H5A···C62	2.8200
O4···H5B^iv^	2.5100	H5A···H62	2.2900
O4···H63^v^	2.8400	H5B···C62	2.8000
O4···H13A	2.5900	H5B···H62	2.3000
O6···H12B^i^	2.7600	H5B···O4^iv^	2.5100
O6···H65^vi^	2.6300	H6···C62^iii^	3.0600
N1···H26	2.8100	H6···C63^iii^	2.7400
N1···H66	2.9000	H6···C64^iii^	3.0500
C12···O6^vii^	3.396 (3)	H6···H2	2.2600
C12···C65^viii^	3.488 (3)	H12A···C65^viii^	2.8100
C13···C22	3.545 (3)	H12A···H66^ii^	2.3900
C16···C35^v^	3.382 (3)	H12B···O6^vii^	2.7600
C16···C34^v^	3.486 (3)	H12B···C16^vii^	3.0800
C22···C13	3.545 (3)	H12B···C25	2.7400
C22···C24^ii^	3.371 (2)	H12B···H25	2.2900
C23···C24^ii^	3.590 (2)	H12C···C25	2.7300
C24···C22^ii^	3.371 (2)	H12C···H25	2.2800
C24···C23^ii^	3.590 (2)	H13A···O4	2.5900
C31···O4	3.082 (2)	H13A···H36	2.3600
C31···C33^ix^	3.468 (2)	H13B···C21	2.5600
C32···C32^ix^	3.553 (2)	H13B···C22	2.8700
C32···C33^ix^	3.540 (3)	H13B···H26^iii^	2.6000
C33···C31^ix^	3.468 (2)	H13B···C33^ix^	3.0500
C33···C32^ix^	3.540 (3)	H13B···C34^ix^	2.9900
C34···C16^v^	3.486 (3)	H16A···C34^v^	3.0900
C35···C16^v^	3.382 (3)	H16A···C35^v^	3.0100
C36···O4	3.355 (2)	H16B···C61^xii^	2.9300
C65···C12^viii^	3.488 (3)	H16B···C62^xii^	3.0600
C3···H32	2.9400	H16B···C63	2.7600
C3···H26	3.0800	H16B···C66^xii^	3.0100
C5···H62	2.5200	H16B···H63	2.3200
C12···H66^ii^	3.0200	H16C···C63	2.7600
C12···H25	2.5100	H16C···H63	2.3100
C16···H12B^i^	3.0800	H16C···C34^v^	3.0300
C16···H63	2.5300	H22···H2	2.3300
C21···H13B	2.5600	H22···H23^x^	2.4200
C22···H23^x^	3.0900	H23···C22^x^	3.0900
C22···H13B	2.8700	H23···H22^x^	2.4200
C23···H34^ix^	2.9100	H25···C12	2.5100
C24···H34^ix^	2.8700	H25···H12B	2.2900
C25···H12B	2.7400	H25···H12C	2.2800
C25···H12C	2.7300	H25···H34^xi^	2.5900
C26···H33^xi^	3.0100	H26···N1	2.8100
C26···H3	2.8900	H26···C3	3.0800
C26···H1	3.05 (2)	H26···H3	2.5500
C32···H3	2.7500	H26···H13B^xii^	2.6000
C33···H13B^ix^	3.0500	H26···H33^xi^	2.5900
C34···H16A^v^	3.0900	H32···C3	2.9400
C34···H16C^v^	3.0300	H32···H3	2.3200
C34···H13B^ix^	2.9900	H33···C26^xi^	3.0100
C35···H16A^v^	3.0100	H33···H26^xi^	2.5900
C61···H16B^iii^	2.9300	H34···C23^ix^	2.9100
C62···H5A	2.8200	H34···C24^ix^	2.8700
C62···H6^xii^	3.0600	H34···H25^xi^	2.5900
C62···H16B^iii^	3.0600	H36···H13A	2.3600
C62···H5B	2.8000	H62···C5	2.5200
C63···H6^xii^	2.7400	H62···H5A	2.2900
C63···H16C	2.7600	H62···H5B	2.3000
C63···H16B	2.7600	H63···C16	2.5300
C64···H6^xii^	3.0500	H63···H16B	2.3200
C65···H12A^viii^	2.8100	H63···H16C	2.3100
C66···H1	2.748 (19)	H63···O4^v^	2.8400
C66···H16B^iii^	3.0100	H65···O6^vi^	2.6300
H1···C26	3.05 (2)	H66···N1	2.9000
H1···C66	2.748 (19)	H66···H1	2.3600
H1···H66	2.3600	H66···O2^ii^	2.8000
H1···O2^ii^	2.56 (2)	H66···C12^ii^	3.0200
H2···H6	2.2600	H66···H12A^ii^	2.3900
H2···H22	2.3300		
			
C12—O2—C24	116.63 (16)	C13—C3—H3	107.00
C16—O6—C64	117.48 (16)	C4—C5—H5A	110.00
C2—N1—C6	111.11 (14)	C4—C5—H5B	110.00
C2—N1—H1	106.0 (11)	C6—C5—H5A	110.00
C6—N1—H1	111.0 (12)	C6—C5—H5B	110.00
N1—C2—C3	108.50 (14)	H5A—C5—H5B	108.00
N1—C2—C21	110.89 (14)	N1—C6—H6	108.00
C3—C2—C21	111.61 (14)	C5—C6—H6	108.00
C2—C3—C4	108.85 (14)	C61—C6—H6	108.00
C4—C3—C13	112.38 (14)	O2—C12—H12A	109.00
C2—C3—C13	112.83 (14)	O2—C12—H12B	109.00
O4—C4—C5	122.03 (15)	O2—C12—H12C	109.00
O4—C4—C3	121.98 (15)	H12A—C12—H12B	109.00
C3—C4—C5	115.96 (14)	H12A—C12—H12C	109.00
C4—C5—C6	109.84 (14)	H12B—C12—H12C	109.00
N1—C6—C5	107.86 (14)	C3—C13—H13A	108.00
C5—C6—C61	113.46 (14)	C3—C13—H13B	108.00
N1—C6—C61	112.45 (14)	C31—C13—H13A	108.00
C3—C13—C31	115.19 (15)	C31—C13—H13B	108.00
C2—C21—C26	121.64 (15)	H13A—C13—H13B	107.00
C22—C21—C26	117.68 (15)	O6—C16—H16A	109.00
C2—C21—C22	120.65 (15)	O6—C16—H16B	109.00
C21—C22—C23	121.49 (16)	O6—C16—H16C	109.00
C22—C23—C24	119.89 (16)	H16A—C16—H16B	109.00
O2—C24—C23	115.62 (15)	H16A—C16—H16C	109.00
C23—C24—C25	119.60 (15)	H16B—C16—H16C	109.00
O2—C24—C25	124.79 (16)	C21—C22—H22	119.00
C24—C25—C26	119.66 (16)	C23—C22—H22	119.00
C21—C26—C25	121.68 (16)	C22—C23—H23	120.00
C32—C31—C36	117.90 (16)	C24—C23—H23	120.00
C13—C31—C32	120.30 (15)	C24—C25—H25	120.00
C13—C31—C36	121.67 (16)	C26—C25—H25	120.00
C31—C32—C33	121.20 (17)	C21—C26—H26	119.00
C32—C33—C34	120.17 (19)	C25—C26—H26	119.00
C33—C34—C35	119.51 (18)	C31—C32—H32	119.00
C34—C35—C36	120.21 (17)	C33—C32—H32	119.00
C31—C36—C35	120.98 (17)	C32—C33—H33	120.00
C62—C61—C66	116.82 (16)	C34—C33—H33	120.00
C6—C61—C62	123.61 (17)	C33—C34—H34	120.00
C6—C61—C66	119.41 (15)	C35—C34—H34	120.00
C61—C62—C63	122.34 (18)	C34—C35—H35	120.00
C62—C63—C64	119.71 (17)	C36—C35—H35	120.00
O6—C64—C65	115.68 (17)	C31—C36—H36	120.00
O6—C64—C63	124.97 (16)	C35—C36—H36	120.00
C63—C64—C65	119.34 (17)	C61—C62—H62	119.00
C64—C65—C66	120.09 (18)	C63—C62—H62	119.00
C61—C66—C65	121.70 (17)	C62—C63—H63	120.00
N1—C2—H2	109.00	C64—C63—H63	120.00
C3—C2—H2	109.00	C64—C65—H65	120.00
C21—C2—H2	109.00	C66—C65—H65	120.00
C2—C3—H3	108.00	C61—C66—H66	119.00
C4—C3—H3	108.00	C65—C66—H66	119.00
			
C12—O2—C24—C23	−179.92 (18)	C3—C13—C31—C32	−59.1 (2)
C12—O2—C24—C25	0.2 (3)	C3—C13—C31—C36	125.18 (18)
C16—O6—C64—C63	0.3 (3)	C2—C21—C22—C23	−178.11 (16)
C16—O6—C64—C65	−179.06 (16)	C26—C21—C22—C23	0.0 (3)
C6—N1—C2—C3	−66.26 (17)	C2—C21—C26—C25	177.96 (16)
C6—N1—C2—C21	170.83 (14)	C22—C21—C26—C25	−0.2 (3)
C2—N1—C6—C5	66.38 (17)	C21—C22—C23—C24	−0.1 (3)
C2—N1—C6—C61	−167.76 (14)	C22—C23—C24—O2	−179.71 (16)
N1—C2—C3—C4	54.73 (17)	C22—C23—C24—C25	0.2 (3)
N1—C2—C3—C13	−179.81 (13)	O2—C24—C25—C26	179.58 (17)
C21—C2—C3—C4	177.22 (14)	C23—C24—C25—C26	−0.3 (3)
C21—C2—C3—C13	−57.32 (19)	C24—C25—C26—C21	0.3 (3)
N1—C2—C21—C22	−128.35 (17)	C13—C31—C32—C33	−175.30 (16)
N1—C2—C21—C26	53.6 (2)	C36—C31—C32—C33	0.6 (3)
C3—C2—C21—C22	110.55 (18)	C13—C31—C36—C35	173.98 (17)
C3—C2—C21—C26	−67.5 (2)	C32—C31—C36—C35	−1.8 (3)
C2—C3—C4—O4	128.46 (18)	C31—C32—C33—C34	1.0 (3)
C2—C3—C4—C5	−49.42 (19)	C32—C33—C34—C35	−1.2 (3)
C13—C3—C4—O4	2.7 (2)	C33—C34—C35—C36	0.0 (3)
C13—C3—C4—C5	−175.14 (15)	C34—C35—C36—C31	1.6 (3)
C2—C3—C13—C31	165.03 (14)	C6—C61—C62—C63	−176.17 (16)
C4—C3—C13—C31	−71.44 (19)	C66—C61—C62—C63	−0.7 (3)
O4—C4—C5—C6	−127.52 (18)	C6—C61—C66—C65	176.14 (17)
C3—C4—C5—C6	50.36 (19)	C62—C61—C66—C65	0.5 (3)
C4—C5—C6—N1	−55.62 (18)	C61—C62—C63—C64	0.4 (3)
C4—C5—C6—C61	179.12 (14)	C62—C63—C64—O6	−179.03 (16)
N1—C6—C61—C62	−122.67 (18)	C62—C63—C64—C65	0.3 (3)
N1—C6—C61—C66	62.0 (2)	O6—C64—C65—C66	178.87 (17)
C5—C6—C61—C62	0.1 (2)	C63—C64—C65—C66	−0.5 (3)
C5—C6—C61—C66	−175.27 (15)	C64—C65—C66—C61	0.1 (3)

Symmetry codes: (i) *x*+1, *y*, *z*−1; (ii) −*x*+1, −*y*, −*z*+1; (iii) *x*−1, *y*, *z*; (iv) −*x*+1, −*y*+1, −*z*; (v) −*x*+2, −*y*+1, −*z*; (vi) −*x*+3, −*y*, −*z*; (vii) *x*−1, *y*, *z*+1; (viii) −*x*+2, −*y*, −*z*+1; (ix) −*x*, −*y*+1, −*z*+1; (x) −*x*, −*y*, −*z*+1; (xi) −*x*+1, −*y*+1, −*z*+1; (xii) *x*+1, *y*, *z*.

### Hydrogen-bond geometry (Å, °)

**Table:**

*D*—H···*A*	*D*—H	H···*A*	*D*···*A*	*D*—H···*A*
N1—H1···O2^ii^	0.92 (2)	2.56 (2)	3.420 (2)	154.9 (14)
C5—H5B···O4^iv^	0.99	2.51	3.490 (2)	173
C16—H16B···Cg1^xii^	0.98	2.85	3.659 (2)	140
C16—H16C···Cg2^v^	0.98	2.98	3.587 (2)	121
C34—H34···Cg3^ix^	0.95	2.91	3.719 (2)	144

Symmetry codes: (ii) −*x*+1, −*y*, −*z*+1; (iv) −*x*+1, −*y*+1, −*z*; (xii) *x*+1, *y*, *z*; (v) −*x*+2, −*y*+1, −*z*; (ix) −*x*, −*y*+1, −*z*+1.
